# Luteal Phase Ovarian Stimulation versus Follicular Phase Ovarian Stimulation results in different Human Cumulus cell genes expression: A pilot study

**DOI:** 10.7150/ijms.55955

**Published:** 2021-02-04

**Authors:** Yu-Chen Chen, Ju-Yueh Li, Chia-Jung Li, Kuan-Hao Tsui, Peng-Hui Wang, Zhi-Hong Wen, Li-Te Lin

**Affiliations:** 1 Department of Obstetrics and Gynecology, Kaohsiung Veterans General Hospital, Kaohsiung City, Taiwan.; 2 Department of Obstetrics and Gynecology, National Yang-Ming University School of Medicine, Taipei City, Taiwan.; 3 Institute of BioPharmaceutical Sciences, National Sun Yat‑sen University, Kaohsiung City, Taiwan.; 4 Department of Obstetrics and Gynecology, Taipei Veterans General Hospital, Taipei City, Taiwan.; 5 Department of Medical Research, China Medical University Hospital, Taichung City, Taiwan.; 6 Department of Marine Biotechnology and Resources, National Sun Yat-sen University, Kaohsiung City, Taiwan.

**Keywords:** cumulus cells, follicular phase ovarian stimulation, gene expression, luteal phase ovarian stimulation, poor ovarian responders

## Abstract

**Background:** Luteal-phase ovarian stimulation (LPOS) is an alternative in vitro fertilization (IVF) protocol. However, limited data showed the genes expression of cumulus cells (CCs) in LPOS. Therefore, this study aimed to investigate CC genes expression between LPOS and follicular-phase ovarian stimulation (FPOS) in poor ovarian responders (PORs) undergoing IVF cycles.

**Methods:** This was a prospective non-randomized trial (ClinicalTrials.gov Identifier: NCT03238833). A total of 36 PORs who met the Bologna criteria and underwent IVF cycles were enrolled. Fifteen PORs were allocated to the LPOS group, and 21 PORs were allocated to the FPOS group. The levels of CC genes involved in inflammation (CXCL1, CXCL3, TNF, PTGES), oxidative phosphorylation (NDUFB7, NDUFA4L2, SLC25A27), apoptosis (DAPK3, BCL6B) and metabolism (PCK1, LDHC) were analyzed using real-time quantitative PCR and compared between the two groups.

**Results:** The number of retrieved oocytes, metaphase II oocytes, fertilized oocytes, day-3 embryos and top-quality day-3 embryos, clinical pregnancy rates and live birth rates were similar between the two groups except for significantly high progesterone levels in the LPOS group. The mRNA expression levels of CXCL1 (0.51 vs 1.00, *p* < 0.001) and PTGES (0.30 vs 1.00, *p* < 0.01) were significantly lower in the LPOS group than in the FPOS group. The LPOS group had significantly lower mRNA expression of NDUFB7 (0.12 vs 1.00, *p* < 0.001) and NDUFA4L2 (0.33 vs 1.00, *p* < 0.01) than the FPOS group. DAPK3 (3.81 vs 1.00, *p* < 0.05) and BCL6B (2.59 vs 1.00, *p* < 0.01) mRNA expression was significantly higher in the LPOS group than in the FPOS group. Increased expression of PCK1 (3.13 vs. 1.00, *p* < 0.001) and decreased expression of LDHC (0.12 vs. 1.00, *p* < 0.001) were observed in the LPOS group compared to the FPOS group.

**Conclusions:** Our data revealed different CC genes expression involving in inflammation, oxidative phosphorylation, apoptosis and metabolism between LPOS and FPOS in PORs. However, the results are non-conclusive; further large-scale randomized controlled trials are needed to validate the results.

## Introduction

Luteal-phase ovarian stimulation (LPOS), referring to the initiation of ovarian stimulation at the luteal phase, has been regarded as a feasible protocol for in vitro fertilization (IVF) cycles 1 based on the previously proposed theory of multiple follicular recruitment waves in the same menstrual cycle 2. LPOS was first applied to fertility preservation in cancer patients 3, 4 and then used in the general population of infertile couples 5, 6. Studies have reported similar numbers of retrieved and mature oocytes and similar rates of fertilization between LPOS and follicular-phase ovarian stimulation (FPOS) in urgent fertility preservation 3, 4 or in women with normal ovarian response 5, 6. Some studies have revealed that in poor ovarian responders (PORs), LPOS could yield more competent oocytes and embryos than FPOS 7-9. The possible rationale was that physiologically high levels of progesterone in the luteal phase could effectively block a premature luteinizing hormone (LH) surge which more frequently occurred in PORs during ovarian stimulation. Our previous study demonstrated that the numbers of retrieved oocytes, metaphase II oocytes, fertilized oocytes, and day-3 embryos were significantly higher in the LPOS group than in the FPOS group 7. However, there have been several studies with conflicting results 10, 11. Furthermore, large-scale randomized controlled trials that validate the results are lacking. Therefore, to date, there is no solid evidence supporting the notion that PORs can truly benefit from LPOS.

Cumulus cells (CCs) are somatic cells that surround the oocyte in cumulus-oocyte complexes (COCs). Bidirectional intercellular communication between CCs and the oocyte is mediated by a network of specialized gap junctions that are crucial for the development of follicles 12. Oocyte-secreted factors, such as growth-differentiation factor 9 (GDF9) and bone morphogenetic protein 15 (BMP15), which are generated from the oocyte, regulate the proliferation, apoptosis, luteinization, metabolism and expansion of CCs 13. CCs protect and nurture the oocyte, playing an essential role in oocyte maturation, ovulation and fertilization 14. Therefore, the expression profiles of CCs have the potential to reflect oocyte competence and even serve as predictors to determine embryo quality, pregnancy and live birth outcomes 15-17.

Although progesterone can prevent premature luteinization effectively 18, 19, the influence of high levels of progesterone on oocytes or CCs is poorly understood. Therefore, the goal of this study was to investigate the differences in human CC genes expression between LPOS and FPOS.

## Materials and Methods

### Study population and design

This prospective non-randomized study was implemented at the Reproductive Medicine Center of the Kaohsiung Veterans General Hospital from August 2017 to December 2018. We enrolled PORs undergoing IVF cycles. The inclusion criteria for POR in this study were defined according to the Bologna criteria 20, and the subjects had at least two of the three following features: (i) advanced maternal age (≥40 years) or any other risk factor for poor ovarian response; (ii) a previous incidence of POR poor ovarian response (≤ 3 oocytes with a conventional stimulation protocol); or (iii) an abnormal ovarian reserve test. In this study, an abnormal ovarian reserve test was defined as an antral follicle count (AFC) < 5 or an anti-Müllerian hormone (AMH) concentration < 1 ng/mL. Furthermore, two episodes of a previous POR after maximal stimulation alone would be sufficient to define a patient as a POR. Patients were excluded if they had any of the following: (i) a diagnosis of primary ovarian insufficiency; (ii) a history of oophorectomy; (iii) a history of exposure to cytotoxic agents or pelvic irradiation for malignancy; or (iv) a history of adjuvant supplementation or hormonal replacement therapy during the previous 3 months. The enrolled participants were then divided into two groups: FPOS or LPOS. The choice of LPOS or FPOS depended on the patients' consideration and preference after full consultation provided by a physician.

### Ethics statement

This study was approved by the institutional review board of Kaohsiung Veterans General (VGHKS15-CT11-12) and Clinical Trial Register (ClinicalTrials.gov Identifier: NCT03238833). All participants were fully counseled, and written informed consent was obtained. This study was in adherence to the approved guidelines and the Declaration of Helsinki.

### Treatment protocol

In the FPOS group, ovarian stimulation with a 300 IU daily dose of combined recombinant follicle-stimulating hormone (rFSH) plus recombinant LH (rLH) (Pergoveris, Merck Serono, Aubonne, Switzerland) commenced within 5 days of the menstrual cycle. In the LPOS group, spontaneous ovulation was confirmed by transvaginal sonography and progesterone levels from day 15 to day 18 of the menstrual cycle. When transvaginal sonography showed absence of dominant follicle and serum progesterone levels were above 1.5 ng/mL, spontaneous ovulation was confirmed. After confirmation of spontaneous ovulation, the women with at least one follicle of less than 8 mm started to undergo ovarian stimulation with a 300 IU daily dose of rFSH plus rLH (Pergoveris, Merck Serono, Aubonne, Switzerland).

In both the FPOS and LPOS groups, when the leading follicle reached 12 mm in diameter, the women received 0.25 mg of GnRH antagonist (Cetrotide; Merck Serono, Idron, France) daily until the day of oocyte trigger. A dual trigger, which comprised a combination of recombinant human chorionic gonadotropin (rHCG) 250 mcg (Ovidrel, Merck Serono, Modugno, Italy) and GnRH agonist 2 mg (Lupro, Nang Kuang Pharmaceutical Co., Ltd., Tainan, Taiwan), was administered when at least one dominant follicle reached 17 mm in diameter. Thirty-six hours after ovulation induction, oocyte retrieval was conducted by transvaginal ultrasound-guided needle aspiration.

Oocytes from all women were inseminated by intracytoplasmic sperm injection (ICSI) to diminish the possibility of fertilization failure. Oocytes were denuded and inseminated if the maturation status was verified by the presence of the first polar body. Fertilization was evaluated 18~20 hours after insemination, and success was defined as the presence of two pronuclei. Embryo development and quality were assessed based on the number and symmetry of the blastomeres and the level of embryonic fragmentation according to the criteria established by the Istanbul consensus workshop 21. All embryos were cryopreserved by vitrification on the third day after oocyte retrieval. An artificial frozen embryo transfer protocol was used for all participants. Oral estradiol (Ediol 8 mg, SynmosaBiopharma Corporation, Hsinchu County, Taiwan) was initiated on the third day of the menstrual cycle, and endometrial thickness was monitored by transvaginal ultrasonography. When the endometrial thickness exceeded 8 mm, luteal support with progesterone, intravaginal gel (crinone 8% gel 90 mg/day, Merck Serono, Hertfordshire, UK) plus oral dydrogesterone (duphaston 40 mg, Abbott, Olst, The Netherlands), was added to oral estradiol. Transabdominal ultrasound-guided embryo transplantation was performed 4 days after commencement of luteal support. The women underwent a pregnancy test 15 days after embryo transfer. If the pregnancy test was positive, oral estradiol was continued until 6 weeks of gestation; progesterone was continued until 8~10 weeks of gestation.

### Cumulus cell collection and gene expression

COCs were collected during oocyte aspiration and washed in the medium. CCs were removed mechanically using a sterile scalpel. CCs separated from the same patient's COCs were pooled together for study. Isolated CCs were then transferred immediately into a sterile tube, centrifuged at 200 g for 5 min at room temperature and stored at -80 °C for further study.

CCs were analyzed for the expression of genes related to inflammation (C-X-C motif chemokine ligand 1 [CXCL1], CXCL3, TNF, prostaglandin E synthase [PTGES]) 22-25, oxidative phosphorylation (NADH ubiquinone oxidoreductase subunit B7 [NDUFB7], NDUFA4 mitochondrial complex associated like 2 [NDUFA4L2], SLC25A27) 26-28, apoptosis (death-associated protein kinase 3 [DAPK3], BCL6B transcription repressor [BCL6B]) 29, 30 and metabolism (phosphoenolpyruvate carboxykinase 1 [PCK1], lactate dehydrogenase C [LDHC]) 31, 32 using real-time quantitative reverse-transcription polymerase chain reaction (qRT-PCR). Gene functions related to reproduction of the included genes are shown in Table S1.

### RNA extraction and real-time qRT-PCR

As previously described 33, total RNA was extracted from CCs with the TRIzol reagent (Invitrogen, Carlsbad, CA, USA) according to the manufacturer's instructions. Each RNA pool was reverse transcribed to cDNA. To detect mRNA expression, qRT-PCR analysis was performed using an ABI Prism 7700 Sequence Detection System (Perkin-Elmer Applied Biosystems, Foster City, CA, USA). PCR was performed using the SYBR Green PCR Core Reagents kit (Perkin-Elmer Applied Biosystems). Gene-specific qRT-PCR primers were used are shown in supplemental Table S2. The thermal cycling conditions included an initial denaturation step at 95 °C for 10 min followed by 40 cycles at 95 °C for 15 s and 60 °C for 1 min. Each set of qRT-PCRs was repeated three times. All of the samples with a coefficient of variation for the Ct value > 1% were retested. GAPDH served as the internal control to normalize the expression of target genes. Relative expression levels were calculated for each sample after normalization to GAPDH. Then, the expression levels of FPOS group was set to be 1 and the relative expression levels of LPOS to the FPOS group was calculated.

### RNA sequencing

Total RNA was extracted from the samples using TRIzol Reagent, according to the manufacturer's instructions. Libraries were prepared using the TruSeq Stranded mRNA LT Sample Prep Kit (Illumina), following the manufacturer's instructions. Differential gene expression analysis was carried out with log_2_ fold change (FC) ≥ 1 or ≤ -1, *p* < 0.05 and false discovery rate (FDR) ≤ 5%.

### Endpoints

The primary endpoint was the mRNA expression of CC genes. Secondary outcome measures included the number of retrieved oocytes, metaphase II oocytes, fertilized oocytes, day-3 embryos and top-quality day-3 embryos, biochemical pregnancy rates, clinical pregnancy rates, miscarriage rates and live birth rates. Clinical pregnancy was defined by the presence of fetal cardiac activity in an intrauterine gestational sac by transvaginal ultrasound. Live birth was determined by delivery of a live fetus after 20 weeks of gestation. Miscarriage refers to pregnancy loss before 20 weeks of gestation.

### Statistical analysis

Parametric t-test or non-parametric Mann-Whitney Wilcoxon methods were used as appropriate for quantitative variables. The categorical variables were compared using chi-square tests or Fisher's exact tests. The statistical analysis was carried out using the Statistical Package for Social Sciences (SPSS) version 22.0 (Chicago, IL, USA) for patients' data and Prism version 6.0 (GraphPad Software, Inc., La Jolla, CA) for genes expression. The differences between groups were considered significant when the *p* value was less than 0.05.

## Results

### Comparison of basic characteristics between FPOS and LPOS groups

A total of 36 patients were recruited in this study and divided into FPOS (n=21) and LPOS (n=15) groups. The baseline characteristics in the two groups are presented in Table 1. The mean age (39.7±3.8 years vs. 40.0±3.4 years) and body mass index (21.7±3.1 kg/m^2^ vs. 23.6±3.8 kg/m^2^) of patients between the two groups were similar. Additionally, there were no statistically significant differences between groups in terms of infertility duration, previous IVF attempts, primary or secondary infertility, infertility causes, basal FSH, AFC and AMH.

### Comparison of cycle characteristics and pregnancy outcomes between FPOS and LPOS groups

The stimulation cycle outcomes of each group are shown in Table 2. No statistically significant difference existed in the duration of stimulation, total dose of gonadotrophins, or peak level of serum estradiol. However, the serum progesterone level on the trigger day was significantly higher in the LPOS group than in the FPOS group (6.8±6.8 ng/ml vs. 0.5±0.2 ng/ml, *p* = 0.004).

No difference between the FPOS and LPOS groups was observed regarding the number of retrieved oocytes (3.0±1.4 vs. 3.1±1.6, *p* = 0.713), metaphase II oocytes (2.2±1.5 vs. 2.3±1.3, *p* = 0.847), fertilized oocytes (1.4±1.1 vs. 2.0±1.3, *p* = 0.207), day-3 embryos (1.5±1.2 vs. 1.9±1.3, *p* = 0.357) or top-quality day-3 embryos (0.4±0.6 vs. 0.7±1.0, *p* = 0.249). Moreover, biochemical pregnancy rates (23.8% vs. 20.0%, *p* = 0.786), clinical pregnancy rates (14.3% vs. 13.3%, *p* = 0.935), live birth rates (9.5% vs. 13.3%, *p* = 0.720) and miscarriage rates (33.3% vs. 0.0%, *p* = 0.361) were similar between the two groups.

### Cumulus cell gene expression between FPOS and LPOS groups

**Figure 1:**
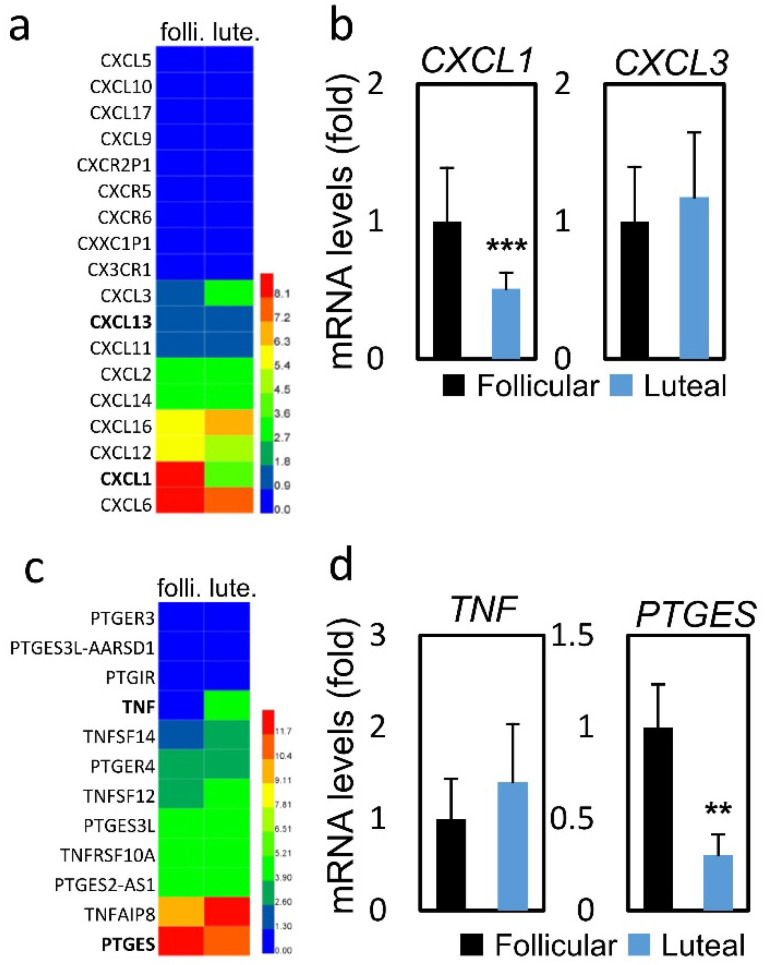
RNA-sequencing data and mRNA levels of cumulus cell genes involved in inflammation in the follicular-phase ovarian stimulation group (Follicular) versus the luteal-phase ovarian stimulation group (Luteal). (a and c) Heat map of RNA-sequencing data. (b and d) mRNA levels of selected inflammation-related genes (CXCL1, CXCL3, TNF, PTGES).

To assess the possible mode of mechanism, we collected follicular and luteal CCs and performed RNA-sequencing (RNA-seq) analysis to assess global changes in transcription. Table 3 presents the mRNA expression of CC genes between FPOS and LPOS groups. The database used for annotation, visualization, and integrated discovery (stacked graph) analysis shows that CXCL1 is down-regulated, while up-regulation of CXCL3 indicates an inflammatory gene cluster, which dominates the structure and housekeeping genes (Figure 1a and 1c). As shown in Figure 1b and 1d, regarding inflammation-related genes, CXCL1 (0.51 vs 1.00, *p* < 0.001) and PTGES (0.30 vs 1.00, *p* < 0.01) mRNA expression levels were significantly lower in the LPOS group than in the FPOS group. However, the mRNA expression levels of CXCL3 and TNF were not significantly different between the two groups.

**Figure 2:**
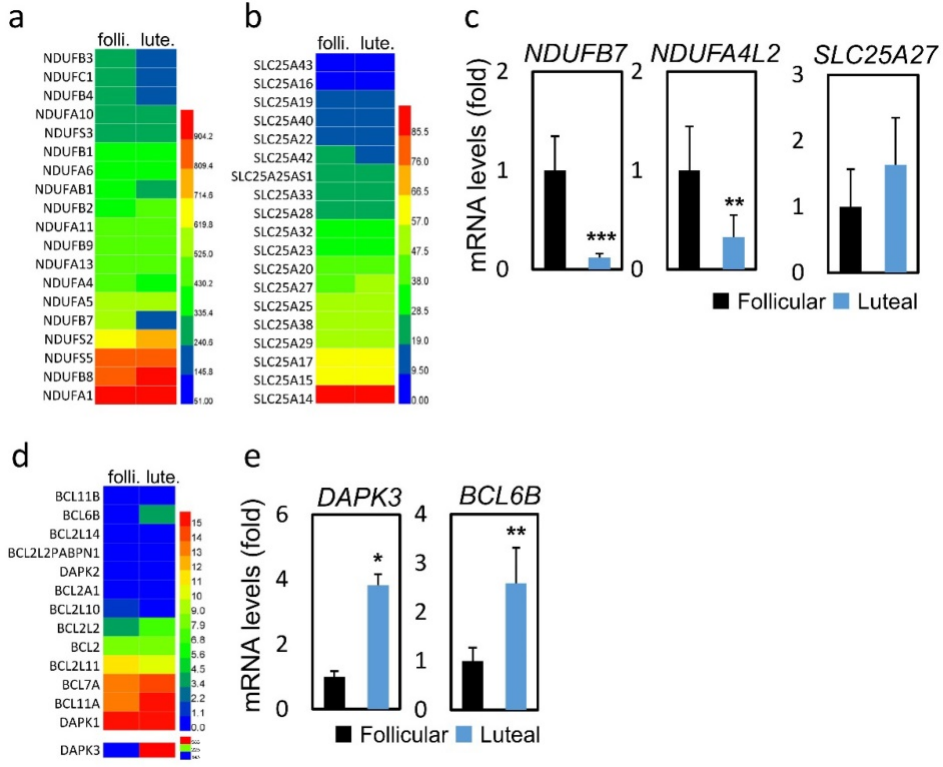
RNA-sequencing data and mRNA levels of cumulus cell genes involved in oxidative phosphorylation or apoptosis in the follicular-phase ovarian stimulation group (Follicular) versus the luteal-phase ovarian stimulation group (Luteal). (a, b and d) Heat map of RNA-sequencing data. (c and e) mRNA levels of selected genes involved in oxidative phosphorylation (NDUFB7, NDUFA4L2, SLC25A27) or apoptosis (DAPK3, BCL6B).

We further analyzed the changes of mitochondrial biogenesis and apoptosis-related genes (Figure 2a, b and d). As shown in Figure 2c and 2e, genes related to oxidative phosphorylation, namely, NDUFB7 (0.12 vs 1.00, *p* < 0.001) and NDUFA4L2 (0.33 vs 1.00, *p* < 0.01) were expressed at lower levels in the LPOS group than in the FPOS group. However, the mRNA expression levels of SLC25A27 were similar between the two groups. In terms of apoptosis-related genes, DAPK3 (3.81 vs 1.00, *p* < 0.05) and BCL6B (2.59 vs 1.00, *p* < 0.01) were more highly expressed in the LPOS group than in the FPOS group.

**Figure 3:**
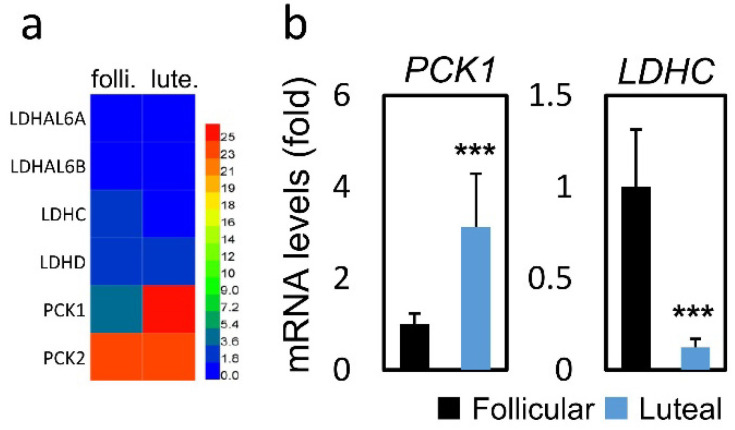
RNA-sequencing data and mRNA levels of cumulus cell genes involved in glucose metabolism in the follicular-phase ovarian stimulation group (Follicular) versus the luteal-phase ovarian stimulation group (Luteal). (a) Heat map of RNA-sequencing data. (b) mRNA levels of selected metabolism-related genes (PCK1, LDHC).

In terms of metabolism-related genes (Figure 3a), compared to the FPOS group, significantly increased PCK1 mRNA expression (3.13 vs. 1.00, *p* < 0.001) and decreased LDHC mRNA expression (0.12 vs. 1.00, *p* < 0.001) were found in the LPOS group (Figure 3b).

## Discussion

To the best of our knowledge, this is the first study to investigate differential mRNA expression in human CCs under LPOS compared to FPOS. This prospective study suggested that in CCs, ovarian stimulation started from the luteal phase or follicular phase might influence the expression of mRNAs related to inflammation, oxidative phosphorylation, apoptosis and metabolism. However, in this study, the number of retrieved oocytes, metaphase II oocytes, day-3 embryos, and top-quality day-3 embryos, as well as the clinical pregnancy and live birth rates, were not significantly different between LPOS and FPOS, mainly due to the small population.

In this study, lower mRNA levels of CXCL1 and PTGES were found in the LPOS group than in the FPOS group. CXCL1, also called interleukin 1 (IL-1), is a member of the CXC subfamily of chemokines. CXCL1 plays a role in inflammation and as a chemoattractant for neutrophils. A prospective study, conducted by Zollner and colleagues and that enrolled 256 couples undergoing IVF/ICSI cycles, showed that high levels of IL-1beta in the follicular fluid were positively associated with fertilization rates 34. Furthermore, a prospective study by Rehman et al. that included a total of 323 patients opting for ICSI demonstrated that higher serum IL-l beta levels were observed in the clinical pregnancy group than in the non-pregnant group or the preclinical abortion group 35. PTGES has three known forms: PTGES1, PTGES2, and PTGES3 36. PTGES is a key enzyme required for the synthesis of PGE2, specifically converting PGH2 to PGE2 37. During the maturation process of bovine oocytes, PTGES, especially PTGES1, works in coordination with PTGS2 to stimulate PGE2 generation 24. During the process of oocyte maturation, PGE2 plays a vital role in cumulus expansion and oocyte meiosis resumption 38. In addition to oocyte maturation, PGE2 has been shown to be a critical mediator in promoting successful fertilization, embryo development and early implantation 39. Thus, LPOS seemed to have detrimental effects on oocyte maturation and embryo development due to reduced IL-1 and PGE2 production.

In this study, the mRNA expression of NDUFB7 and NDUFA4L2 was significantly lower in the LPOS group than in the FPOS group. NDUFB7 and NDUFA4L2 encode a protein involved in the electron transport chain (ETC), which is the main process of ATP production in the mitochondria. The complex I (NADH: ubiquinone oxidoreductase) is the first enzyme of the mitochondrial respiratory chain and consists of 45 subunits in humans, making it one of the largest known multi-subunit membrane protein complexes 40. Complex I is the first step of the ETC leading to energy production in mitochondria. Abnormalities in the mitochondrial complex I subunit leads to structural breakdown and failure to initiate proton transport, thus preventing successful ATP production. Once protons are leaked and fail to propagate to other complexes, they cause imbalance mitochondrial membrane potential and increased oxidative stress, which also induces the production of various inflammatory factors, leading to cellular senescence and cell death 41. Numerous studies have indicated that mitochondrial dysfunction in oocytes has negative impacts on oocyte maturation, fertilization, embryo development, and pregnancy 42-44. Additionally, this study showed that the LPOS group showed higher mRNA expression of DAPK3 and BCL6B than the FPOS group. DAPK3 and BCL6B both play a role in the induction of apoptosis. Increased apoptosis of CCs has been reported to be poorly associated with oocyte maturation, fertilization, embryo development, and pregnancy 45-47. Accordingly, this study seemed to reveal that LPOS may lead to mitochondrial dysfunction and increased apoptosis of CCs, causing adverse reproductive outcomes.

Moreover, in this study, increased mRNA expression of PCK1 and decreased mRNA expression of LDHC were observed in the LPOS group compared to the FPOS group. PCK1 is a central regulator of gluconeogenesis and is regulated by Cited2. Fang et al. demonstrated that high Cited2 protein levels in CCs significantly increased the expression of PCK1 mRNA and the levels of glucose in CCs. It was suggested that the high Cited2 level might impair oocyte quality by up-regulating PCK1 mRNA expression to result in abnormal glucose metabolism in CCs 48. Therefore, the increased mRNA expression of PCK1 in the LPOS group seemed to have an unfavorable influence on oocytes by disrupting glucose metabolism. LDHC catalyzes the conversion of L-lactate and NAD to pyruvate and NADH in the final step of anaerobic glycolysis. Enhanced glucose metabolism via increased glycolysis activity in oocytes is positively associated with oocyte maturation and embryo development 49-51. Hence, LPOS seemed to decrease oocyte competence and embryo growth by decreasing LDHC mRNA expression.

Taken together, the findings of the present study showed that LPOS may diminish IL-1 and PGE2 production, reduce mitochondrial function, increase apoptosis, increase gluconeogenesis and decrease glycolysis in CCs, implying that LPOS might have detrimental effects on CCs. However, a prospective observational study including 39 patients with reduced ovarian reserve undergoing double stimulation showed that significantly higher mRNA levels of VCAN, SDC4, and TP53I3 in CCs was observed in luteal-phase-derived oocytes compared to follicular-phase-derived oocytes 52. The study conducted by Cimadomo et al. revealed similar miRNAs expression between follicular fluids collected after FPOS and paired LPOS in 15 old women with reduced ovarian reserve undergoing double stimulation 53.

Similar IVF outcomes were observed between the LPOS and FPOS groups in this study perhaps due to the non-conclusive results and a small population. Although a previous report proposed that PORs, more likely experiencing breakthrough premature LH surge under GnRH antagonist protocol 54, may benefit from LPOS because the physiologically elevated progesterone levels associated with LPOS could prevent a premature LH rise naturally in the luteal phase 7, there was no definite clinical evidence to support this idea. Some studies revealed that LPOS increased the likelihood of gaining more competent oocytes and embryos in PORs compared with FPOS 7-9. However, these studies were not randomized controlled trials and had small numbers of patients. A randomized controlled pilot trial performed by Kansal Kalra and colleagues revealed that IVF outcomes, including the number of oocytes retrieved, number of embryos transferred, clinical pregnancy rate and live birth rate, of LPOS and FPOS were similar in PORs 10. Another updated randomized controlled pilot trial showed that LPOS was comparable with FPOS in terms of the number of MII oocytes, but had higher ovarian responsiveness than FPOS in PORs 55. Additionally, a retrospective study conducted by Wu et al., including 274 PORs, suggested that there was no significant difference regarding the mean number of retrieved oocytes, the mean number of embryos, the implantation rate or the clinical pregnancy rate between LPOS and FPOS 11, and these results are similar to those of the current study, despite the small population of the current study. Admittedly, progestins have been proven to be able to inhibit an early-onset LH surge effectively, but the effects of high levels of progesterone on oocytes or CCs remain unclear. This study showed that LPOS might have harmful effects on CCs in PORs. However, large-scale randomized controlled trials are required to confirm the results of this study.

For the interpretation of the data in this study, several limitations should be taken into account. First, this was a non-randomized trial and had a small study population. Thus, the results are non-conclusive. Future large-scale randomized controlled trials are required to confirm the results. Second, the group of participants, who were enrolled based on the Bologna criteria, may be heterogeneous. Third, a limited number of CC genes were analyzed in this study. Therefore, the conclusion is not fully supported by the result of the present study. Forth, the changes of protein expression were not validated in this study due to limited amount of samples. Hence, the results are not truly reliable. However, the strengths of this study were that all the IVF protocols were carried out by the same physician and that all the laboratory procedures were executed by the same embryologist, which minimize the bias in performance.

In conclusion, this study showed that LPOS might have a disadvantageous influence on CCs in PORs via decreased expression of CXCL1, PTGES, NDUFB7, NDUFA4L2, and LDHC and increased expression of DAPK3, BCL6B, and PCK1, implying that LPOS seemed to decrease beneficial inflammation and mitochondrial function and augment apoptosis and abnormal glucose metabolism in CCs. However, the results are non-conclusive. Further randomized controlled trials with large populations are needed to verify these results.

## Supplementary Material

Supplementary table S1.

## Figures and Tables

**Table 1:** Basic characteristics of poor ovarian responders undergoing follicular-phase ovarian stimulation (FPOS) or luteal-phase ovarian stimulation (LPOS)

Parameters	FPOS (n=21)	LPOS (n=15)	*p* value
Age (years)	39.7±3.8	40.0±3.4	0.818
Body mass index (kg/m^2^)	21.7±3.1	23.6±3.8	0.128
Infertility duration (years)	5.1±3.2	5.3±6.6	0.882
Previous IVF attempts (n)	2.9±2.9	2.1±2.6	0.441
**Types of infertility (%)**			0.204
Primary infertility	52.4 (11/21)	73.3 (11/15)	
Secondary infertility	47.6 (10/21)	26.7 (4/15)	
**Infertility causes (%)**			0.970
Tubal factor	9.5 (2/21)	13.3 (2/15)	
Male factor	14.3 (3/15)	13.3 (2/15)	
Endometriosis	19.0 (4/15)	26.7 (4/15)	
Uterine factor	23.8 (5/15)	20.0 (3/15)	
Multiple factors	33.3 (7/15)	26.7 (4/15)	
Basal FSH (IU/l)	5.6±4.0	5.4±2.2	0.864
Antral follicle counts (n)	4.4±1.4	4.9±1.7	0.273
Anti-Müllerian hormone (ng/ml)	0.7±0.4	0.7±0.6	0.441

Data are presented as the mean ± standard deviation or percentage. IVF, *in vitro* fertilization; FSH, follicle-stimulating hormone.

**Table 2:** Cycle characteristics and pregnancy outcomes of poor ovarian responders undergoing follicular-phase ovarian stimulation (FPOS) or luteal-phase ovarian stimulation (LPOS)

Parameters	FPOS (n=21)	LPOS (n=15)	*p* value
Stimulation duration (days)	11.3±2.2	12.1±2.8	0.363
Gonadotropin dosage (IU)	2882.1±690.1	2885.0±744.0	0.991
E2 on the trigger day (pg/mL)	749.9±553.2	756.7±671.5	0.975
P on the trigger day (ng/mL)	0.5±0.2	6.8±6.8	0.004
No. of oocytes retrieved (n)	3.0±1.4	3.1±1.6	0.713
No. of metaphase II oocytes (n)	2.2±1.5	2.3±1.3	0.847
Maturation rate (%)	69.1±36.7	72.9±28.5	0.742
No. of fertilized oocytes (n)	1.4±1.1	2.0±1.3	0.207
Fertilization rate (%)	61.7±41.1	79.6±32.2	0.170
No. of Day 3 embryos (n)	1.5±1.2	1.9±1.3	0.357
No. of top-quality Day 3 embryos (n)	0.4±0.6	0.7±1.0	0.249
Biochemical pregnancy rate (%)	23.8 (5/21)	20.0 (3/15)	0.786
Clinical pregnancy rate (%)	14.3 (3/21)	13.3 (2/15)	0.935
Live birth rate (%)	9.5 (2/21)	13.3 (2/15)	0.720
Miscarriage rate (%)	33.3 (1/3)	0.0 (0/2)	0.361

Data are presented as the mean ± standard deviation or percentage. E2, estradiol; P, progesterone.

**Table 3:** Cumulus cell genes mRNA expression in follicular-phase ovarian stimulation (FPOS) and luteal-phase ovarian stimulation (LPOS) groups

Gene name	FPOS (n=21)		LPOS (n=15)		*p* value
	Original^*^	Standardized^#^	Original^*^	Standardized^#^	
CXCL1	1.17±0.46	1	0.60±0.16	0.51	< 0.001
CXCL3	0.10±0.04	1	0.11±0.05	1.18	0.963
TNF	0.08±0.03	1	0.11±0.05	1.40	0.404
PTGES	0.72±0.17	1	0.22±0.08	0.30	0.001
NDUFB7	2.52±0.88	1	0.29±0.11	0.12	< 0.001
NDUFA4L2	0.50±0.22	1	0.16±0.11	0.33	0.002
SLC25A27	0.09±0.05	1	0.14±0.06	1.64	0.597
DAPK3	0.91±0.16	1	3.49±0.32	3.81	0.032
BCL6B	0.02±0.01	1	0.06±0.02	2.59	0.001
PCK1	0.15±0.04	1	0.48±0.18	3.12	< 0.001
LDHC	17.49±5.50	1	2.19±0.81	0.12	< 0.001

*Original data are a fold change with respect to the normalization control gene GAPDH and are presented as the mean ± standard error of the mean. #Regarding standardized data, the data in the FPOS group were set up to be 1 and those in the LPOS group were the ratio of LPOS/FPOS.

## Abbreviations

AFC: antral follicle count
AMH: anti-Müllerian hormone
BCL6B: BCL6B transcription repressor
BMP15: bone morphogenetic protein 15
CC: cumulus cell
COC: cumulus-oocyte-complex
CXCL1: C-X-C motif chemokine ligand 1
DAPK3: death-associated protein kinase 3
FPOS: follicular-phase ovarian stimulation
FSH: follicle-stimulating hormone
GDF9: growth-differentiation factor 9
ICSI: intracytoplasmic sperm injection
IL-1: interleukin 1
IVF: *in vitro* fertilization
LDHC: lactate dehydrogenase C
LH: luteinizing hormone
LPOS: luteal-phase ovarian stimulation
NDUFA4L2: NDUFA4 mitochondrial complex associated like 2
NDUFB7: NADH ubiquinone oxidoreductase subunit B7
PCK1: phosphoenolpyruvate carboxykinase 1
POR: poor ovarian responder
PTGES: prostaglandin E synthase
qRT-PCR: real-time quantitative reverse-transcription polymerase chain reaction
rFSH: recombinant follicle-stimulating hormone
rHCG: recombinant human chorionic gonadotropin
