# Supplementary material for: Luteal Phase Ovarian Stimulation versus Follicular Phase Ovarian Stimulation results in different Human Cumulus cell genes expression: A pilot study
Source: Int J Med Sci. 2021 Feb 4;18(7):1600–8. doi: 10.7150/ijms.55955 (PMC7976567; doi:10.7150/ijms.55955)
Supplement: Supplementary file 1 — Supplementary table S1. [file ijmsv18p1600s1.pdf]

Table S1 Gene function related to reproduction of the included genes

Gene name	Gene function related to reproduction	Ref.
CXCL1	oocyte nuclear maturation	[22]
CXCL3	oocyte nuclear maturation	[22]
TNF	regulation of apoptosis in oocytes and granulosa cells	[23]
PTGES	ovulation, fertilization and embryo development	[24,25]
NDUFB7	mitochondrial function in oocytes and embryos¯	[26]
NDUFA4L2	electron transport chain and AMH signal transduction	[27]
SLC25A27	oocyte maturation and embryo development	[28]
DAPK3	androgen receptor-mediated transcription	[29]
BCL6B	embryogenesis	[30]
PCK1	glucose homeostasis in cumulus cells	[31]
LDHC	glucose metabolism in oocytes	[32]

Table S2 Primers used for real time polymerase chain reaction analysis

Gene name	Primers		Genbank accession no.
GAPDH	Forward primer:	CGACCACTTTGTCAAGCTCA	NM_002046
	Reverse primer:	AGGGGAGATTCAGTGTGGTG	
CXCL1	Forward primer:	ATTCACCCCAAGAACATCCA	NM_001511
	Reverse primer:	CACCAGTGAGCTTCCTCCTC	
CXCL3	Forward primer:	GCAGGGAATTCACCTCAAGA	NM_002090
	Reverse primer:	ACCCTGCAGGAAGTGTCAAT	
TNF	Forward primer:	CCTGTGAGGAGGACGAACAT	NM_000594
	Reverse primer:	AGGCCCCAGTTTGAATTCTT	
PTGES	Forward primer:	CATGTGAGTCCCTGTGATGG	NM_004878
	Reverse primer:	CTGCAGCAAAGACATCCAAA	
NDUFB7	Forward primer:	CTGACTGAGGGGTCAGTGGT	NM_004146
	Reverse primer:	CCATCATCTCCTGCTGTGTG	
NDUFA4L2	Forward primer:	TCGGAGAGCAGAAACCACTT	NM_020142
	Reverse primer:	TTTCCCAGTCTGGTCCTCTG	
SLC25A27	Forward primer:	GCGAGAAGGAGTGCGTTATC	NM_004277
	Reverse primer:	GCTTCTCCTTGCATTTGGAG	
DAPK3	Forward primer:	TCGCACACTTTGACCTGAAG	NM_001348
	Reverse primer:	CAGCGGCTCATAGTTCACAA	
BCL6B	Forward primer:	GCCCCTTCTTCCTTTAGTGG	NM_181844
	Reverse primer:	AATGGAACTCAGCCCCTTTT	
PCK1	Forward primer:	GGGAGAAGGAGGTGGAAGAC	NM_002591
	Reverse primer:	GAACACTTGCCCTCTCTTGC	
LDHC	Forward primer:	TCTGTACTGATTGCGCCAAG	NM_002301
	Reverse primer:	GGCAAGTTCATCAGCCAAAT	
